# Pilar leiomyoma with dermatofibroma-like dermatoscopic features: a clinical challenge

**DOI:** 10.1093/skinhd/vzag088

**Published:** 2026-06-23

**Authors:** Grigorios Theodosiou

**Affiliations:** Department of Clinical Sciences, University of Lund, Lund, Sweden; Department of Dermatology, University Hospital of Skåne, Malmo, Sweden

## Abstract

Pilar leiomyoma is a rare benign tumour that derives from the arrector pili muscle. Due to its rarity, the dermatoscopic features of pilar leiomyoma are scarcely reported in the literature. Herein, we report a further case of pilar leiomyoma that highlights its dermatofibroma-like dermatoscopic features and underscores that, despite advances in noninvasive imaging technologies, histopathology remains the gold standard for the diagnosis of painful skin lesions.

## Teaser

The dermatoscopic features of pilar leiomyoma are poorly investigated due to its rarity. Occasionally, pilar leiomyoma may show dermatofibroma-like features in dermatoscopy with the potential clinical complication of missing or delayed diagnosis of Reed syndrome. Despite the recent advances in noninvasive imaging technologies, high clinical suspicion is critical and histopathology remains the gold standard for the diagnosis of painful skin lesions.

What is already known about this topic?

- Pilar leiomyoma is a rare benign cutaneous tumour originating from the arrector pili muscle.
- Dermoscopic features of pilar leiomyoma are infrequently described in the literature because of its rarity.
- Painful cutaneous lesions often present diagnostic challenges, despite advances in noninvasive imaging techniques.

What does this study add?

- This case further highlights the dermatofibroma-like dermoscopic features that may be observed in pilar leiomyoma.
- The report emphasizes that histopathological examination remains the gold standard for the diagnosis of painful skin lesions.

## Case report

**Figure 1:**
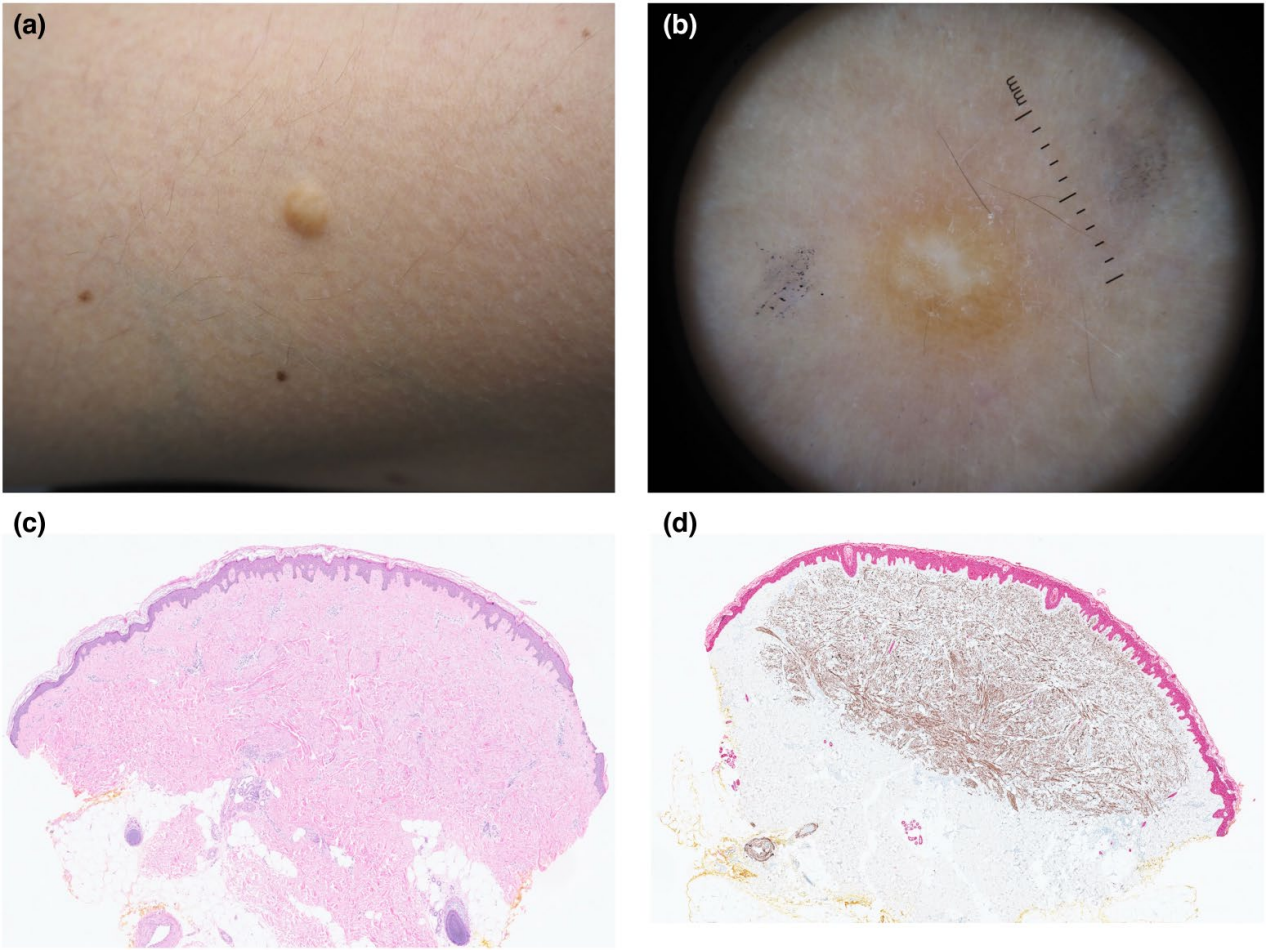
(a) A solitary yellowish nodule. (b) Chalk-white structureless area in the centre of the lesion surrounded by light-brown reticular lines. (c) Interlacing bundles of smooth muscle cells are seen in the dermis (haematoxylin and eosin ×40). (d) Strong and diffuse staining for desmin (magnification ×40).

A 40-year-old white woman was referred to the outpatient clinic of our department with a new-onset skin-coloured nodule on her right leg. The lesion was painful when touched, especially after exposure to cold temperature. Skin examination showed a solitary yellowish nodule, 10 × 12 mm in diameter. The lesion was sensitive on palpation. Dermatoscopy showed a chalk-white structureless area in the centre of the lesion surrounded by light-brown reticular lines, suggesting a diagnosis of dermatofibroma (Figure 1a, b).

Given the discrepancy between the history, symptomatology and the dermatoscopical findings we decided to perform an excisional biopsy of the lesion; the subsequent histopathological examination showed a proliferation located in the papillary and reticular dermis consisting of interlacing bundles of ­spindle-shaped cells with eosinophilic cytoplasm and oval nuclei. A strong and diffuse staining for desmin was seen on immunochemistry (Figure 1c, d).

The lesion was completely excised with clear margins, and the patient is currently on yearly follow-up. With respect to screening, given the solitary nature of the lesion and the absence of a family history suggestive of Reed syndrome (hereditary leiomyomatosis and renal cell carcinoma), genetic screening was not pursued in this instance.

## Discussion

Pilar leiomyomas are rare benign smooth muscle tumours deriving from the arrector pili muscle of the pilosebaceous unit.^1^ Pilar leiomyomas manifest more often as multiple than solitary lesions.^1,2^ Multiple leiomyomas may cluster to form plaques or may be distributed in a zosteriform pattern.^1–3^ Reed syndrome (leiomyomatosis cutis et uteri; OMIM 150800) is a rare autosomal dominant syndrome caused by a germline mutation in *FH*.^2,3^ Reed syndrome is associated with multiple cutaneous and uterine leiomyomas, and increased risk for papillary renal cell carcinoma and renal collecting duct carcinoma.^3^ The cutaneous leiomyomas present in the second or third decade of life and are usually the first manifestation of the syndrome.^1–3^ While cutaneous leiomyomas are the earliest sign of the syndrome, they are often missed or misdiagnosed as benign cutaneous tumours, resulting in a delayed diagnosis of Reed syndrome and a missed window of opportunity to initiate a potentially life-saving screening for renal cell carcinoma.^2,3^

The typical lesion is a skin-coloured or reddish-brownish dermal papule or nodule, up to 2.0 cm in diameter.^1,4^ Multiple ­leiomyomas are commonly localized on the trunk, face and the extensor surface of extremities, distributed in a linear, clustered, segmental or disseminated pattern.^1,4,5^ Minor trauma or exposure to cold temperatures may lead to severe pain in the tumours. Pseudo-Darier sign, an evoked transient piloerection and erythema without pruritus after gentle stoking on the cutaneous leiomyomas, has been described in a few cases.^1,4,5^ The differential diagnosis includes painful skin tumours such as spiradenoma, neurofibroma, angiolipoma, cutaneous endometriosis and glomus tumour, as well as dermatofibroma, smooth muscle cell hamartoma and eccrine poroma.^1,4,5^

Pilar leiomyomas are circumscribed nonencapsulated tumours on the dermis. An overlying zone of uninvolved subepidermal tissue is usually present, and there may be some flattening of the epidermis. Epidermal hyperplasia has also been described as a frequent finding in a case series. The tumour is composed of bundles of smooth muscle arranged in an inter­lacing and sometimes whorled pattern. The smooth muscle nature of the cells can be confirmed with Masson trichrome stain or the use of vimentin, smooth muscle actin, desmin and caldesmon.^1,4–6^

Dermatoscopy of pilar leiomyoma shows features similar to those of dermatofibroma, namely a central hypopigmented structureless area and a delicate peripheral pigmented network. Some lesions also show a circular or elongated hyperpigmented structure within the central whitish area, probably due to scratching.^5–7^

This case illustrates the clinical challenge of managing newly growing painful and sensitive papules and nodules. Even though the dermatoscopic findings of the lesion were suggestive of dermatofibroma, the pain reported by the patient, as well as the sensitivity upon palpation, helped us not to miss the diagnosis of leiomyoma. Despite the recent advances in noninvasive imaging technologies, histopathology remains the gold stadard for the diagnosis of painful skin lesions.
