# Correction to: Pilar leiomyoma with dermatofibroma-like dermatoscopic features: a clinical challenge

**DOI:** 10.1093/skinhd/vzag132

**Published:** 2026-07-29

This is a correction to: Grigorios Theodosiou, Pilar leiomyoma with dermatofibroma-like dermatoscopic features: a clinical challenge, *Skin Health and Disease*, Volume 6, Issue 4, August 2026, Pages 613–615, https://doi.org/10.1093/skinhd/vzag088

The following change has been made to the originally published paper.

In section **Case Report**, the beginning of first sentence has been corrected to read: “A 40-year-old white woman[…]”.

